# Factors associated with exclusive breastfeeding in the first six months of life in Brazil: a systematic review

**DOI:** 10.1590/S0034-8910.2015049005971

**Published:** 2015-12-16

**Authors:** Cristiano Siqueira Boccolini, Márcia Lazaro de Carvalho, Maria Inês Couto de Oliveira

**Affiliations:** I Laboratório de Informação em Saúde. Instituto de Comunicação e Informação Científica e Tecnológica em Saúde. Fundação Oswaldo Cruz. Rio de Janeiro, RJ, Brasil; II Departamento de Epidemiologia e Métodos Quantitativos em Saúde. Escola Nacional de Saúde Pública. Fundação Oswaldo Cruz. Rio de Janeiro, RJ, Brasil; III Departamento de Epidemiologia e Bioestatística. Instituto de Saúde Coletiva. Universidade Federal Fluminense. Niterói, RJ, Brasil

**Keywords:** Breast Feeding, Maternal Behavior, Risk Factors, Socioeconomic Factors, Review

## Abstract

**OBJECTIVE:**

To identify factors associated with exclusive breastfeeding in the first six months of life in Brazil.

**METHODS:**

Systematic review of epidemiological studies conducted in Brazil with exclusive breastfeeding as outcome. Medline and LILACS databases were used. After the selection of articles, a hierarchical theoretical model was proposed according to the proximity of the variable to the outcome.

**RESULTS:**

Of the 67 articles identified, we selected 20 cross-sectional studies and seven cohort studies, conducted between 1998 and 2010, comprising 77,866 children. We identified 36 factors associated with exclusive breastfeeding, being more often associated the distal factors: place of residence, maternal age and education, and the proximal factors: maternal labor, age of the child, use of a pacifier, and financing of primary health care.

**CONCLUSIONS:**

The theoretical model developed may contribute to future research, and factors associated with exclusive breastfeeding may subsidize public policies on health and nutrition.

## INTRODUCTION

Breastfeeding is a crucial issue for public health, for it directly affects the standards of health and mortality of populations.^4,14,20,39,53^ The prevalence and duration of partial or exclusive breastfeeding increased in all social strata and regions of Brazil between the decades of 1990 and 2010.^35,45,a,b^ Part of this trend can be attributed to national policies of breastfeeding promotion, protection, and support.^35^

Different social and cultural contexts may influence the practice of exclusive breastfeeding and its determinants. A study conducted in cities of three countries noted that higher levels of maternal education were related both with higher prevalence of exclusive breastfeeding in Santos, SP, Southeastern Brazil, and with lower prevalence in Mexico City, Mexico, and in Sula and Tegucigalpa, Honduras.^32^

Seeking greater population homogeneity, this review was restricted to the Brazilian context, since the determinants of exclusive breastfeeding may behave differently in diverse cultures.

The aim of this study was to identify the factors associated with exclusive breastfeeding in the first six months of life in Brazil.

## METHODS

Publications of epidemiological studies conducted in Brazil about factors associated with exclusive breastfeeding were analized. Bibliographical research was carried out on Medline (via PubMed) and LILACS databases. Delimitation by period or by language were not considered. A manual search of the references included in the bibliography of each article was carried out.

The searches were independently conducted in July 2014 by two reviewers. The advanced search terms for the PubMed were: (exclusive[All Fields] AND (“breast feeding” [MeSH Terms] OR (“breast”[All Fields] AND “feeding”[All Fields]) OR “breast feeding”[All Fields] OR “breastfeeding”[All Fields]) AND (“Brazil”[MeSH Terms] OR “Brazil”[All Fields])) AND (determinants[All Fields] OR factors[All Fields] OR (“epidemiology”[Subheading] OR “epidemiology”[All Fields] OR “epidemiology”[MeSH Terms]))”.

In the LILACS database were searched, in the “search details”, the following terms: “tw:(tw: (exclusive AND breastfeeding (epidemiology OR determinants OR factors)) AND (instance:“regional”) AND (db:(“LILACS”))) AND (instance:“regional”) AND (mj:(“Breastfeeding”))”. The terms were also used in Portuguese: “tw:(aleitamento AND materno AND exclusivo) AND (instance:“regional”) AND (db:(“LILACS”) AND mj:(“Aleitamento materno”)) OR fatores)) AND (instance:“regional”) AND (db:(“LILACS”))”. The term “Brazil” was not employed in this database, since it has only articles published in journals of Latin America and Caribbean.

Observational analytical epidemiological studies were included, in which exclusive breastfeeding was treated as outcome, with adjustment of the factors studied together and for possible confounding factors, which have adopted the definition of the World Health Organization (WHO)^c^ for exclusive breastfeeding (children receive only human milk, directly from their mother or extracted, and no longer receive any other liquid or solid, except vitamin drops or syrups, vitamin supplements or medication) and whose sampling procedure has generated a representative population of infants from maternities, cities, states, or of the Brazilian nation.

In articles which more than one age group was evaluated (more than a statistical model to evaluate two or more different age groups), the age group of older age was chosen (with a limit of six months), since the objective was to evaluate the outcome closer to the WHO^c^ recommendation of exclusive breastfeeding until six months of age.

The following studies were excluded: those with results subject to selection bias (such as losses exceeding 20.0%) or with possible information bias (such as interviews with mothers of children over one year old); that presented only the p value (without presenting the association measures), which considered only the population born with low weight; and bibliographic reviews (systematic or unsystematic).

In the case of studies using the same database and published in more than one article (different journals and years), we included those that used different age groups or different analytical variables and methods.

The selected articles were stored under the Portable Document Format (PDF) into a directory shared in the cloud, separated according to databases of origin (Medline and LILACS), and classified into different folders between included and excluded.

The evaluation of the methodological quality of the selected studies was obtained by adjusting the scale *“Effective Public Health Practice Project: Quality Assessment Tool for Quantitative Studies – *QATQS*”* (http://www.ephpp.ca/tools.html). Of this scale, five questions were evaluated (classified as “strong”, “moderate”, or “weak”): 1) selection bias; 2) study design; 3) confounding factors; 4) data collection methods; and 5) type of analysis employed for the outcome. Blinding issues of QATQS were not used in any study (since no clinical trial was included), and issues of loss of follow-up were not applied in sectional studies. In the “study design” question, sectional studies had lower score than the cohort studies, because in sectional studies the temporality between exposition variables and the outcome may not always be established.

Considering the final score of the QATQS scale of each article selected, the articles were considered strong if none of the questions were rated as weak; moderate, in the case of studies that showed one of the questions rated as weak; and weak, studies with one or more questions thus evaluated.

Data extraction was independently performed by two reviewers by a structured form, in which were recorded: the last name of the first author; publishing journal and year; location(s) of performance; year and period of performance; study design; population of the study; sample design used; strategy for selection of subjects of research; inclusion and exclusion criteria; total sample number; sample number evaluated; total losses and reason of losses; age group of the studied children; type of outcome; type of statistical analysis; factors of control or adjusting the statistical model; results of the model with association measure and statistical significance; exclusive breastfeeding prevalence or median; limitations of the study; and observations. In the event of disagreement among peers, a third reviewer was consulted.

Data tabulation included: reference of the article (with the last name of the first author, journal, and year of publication); location of the study and data collection; sample number evaluated (and data source); outcome of the study (exclusive breastfeeding or its interruption); statistical analysis employed; prevalence (or median) of exclusive breastfeeding found and the age group of this prevalence (expressed in months); factors associated with exclusive breastfeeding in a statistically significant manner (obtained from the results of the statistical models), as well as its association measure and other factors evaluated without statistically significant association with exclusive breastfeeding.

Two tables were made, one for cross-sectional studies and another for cohort studies. As the revised studies measured the prevalence or the duration of exclusive breastfeeding in different age groups, the summary tables of this outcome contain this information.

The next step consisted in individually analyzing the association found between the factors investigated and exclusive breastfeeding, highlighting and quantifying the following aspects: in how many studies these factors were investigated, in how many studies an association with exclusive breastfeeding was identified in statistical models and what is its direction.

The last step of the study consisted in creating a hierarchical theoretical model (using the assumptions established by Víctora et al)^49^ organizing all factors found according to the proximity to the outcome. The selection of the allocation levels of variables followed the logic of chronological classification between factors present before pregnancy, during pregnancy, immediate postpartum and at the time of discharge until six months of life.

Four levels of variables were proposed, grouped in hierarchical blocks: 1) distal characteristics (contextual, domestic, household, and maternal); 2) distal intermediate (from pregnancy and prenatal care); 3) proximal intermediate (childbirth care, maternal characteristics during hospitalization, and characteristics of the newborn); 4) proximal (characteristics of the nursing mothers and the family, of the babies, and of health services).

To provide parsimony to the summary of variables identified and also for the creation of the theoretical model, the terminology used in each article for each variable was standardized.

## RESULTS

**Figure 1:**
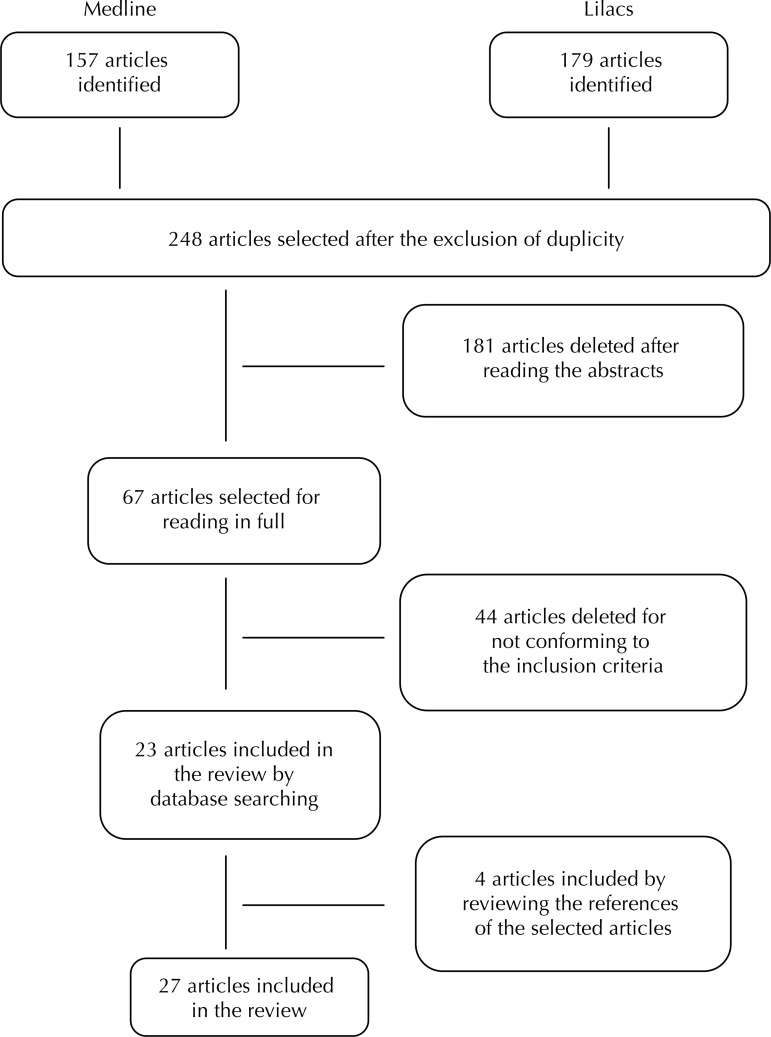
Descriptive flowchart of steps of systematic review in Medline and LILACS databases.

Of the 67 articles retrieved by electronic search, 44 were excluded because they did not meet the selection criteria. After manual search, four articles were included,^21,29,38,44^ totaling 27 articles selected for the analysis^5-7,9-11,15,16,18,21,23,24,28-31,34,39,40,43-47,51,52^ (Figure 1), of which seven are cohort studies and 20 are sectional studies. Of these 20, 12 used questionnaires based on the Breastfeeding and Municipalities Project (AMAMUNIC).^48^ Considering the classification of the articles selected according to the adapted QATQS scale, of the sectional studies 14 were considered moderate, and six, weak (Table 1). Among the cohort studies, six were considered strong, and only one weak (Table 2).

**Table 1:** Sectional studies on factors associated with exclusive breastfeeding.

Author/Publishing year	Local/year of implementation	Sample (n)/data source	Statistical analysis	Quality Score	EBF Prevalence (age group)	Outcome	Factors associated with outcome and association measure	Factors evaluated without statistical significance
Alves et al^1^ (2013)	Barra Mansa, RJ, 2006	707 babies < 6 m/AMAMUNIC	Hierarchical Poisson Regression	Moderate	46.7% (< 6 m)	EBF	Mother’s education ≤ 8^th^ grade [PR = 0.80] Childbirth type (caesarean) [PR = 0.84] Child’s age in days [PR = 0.99] Use of a pacifier [PR = 059] Assistance by a Breastfeeding-Friendly Primary Care Unit [PR = 1.19]	Adolescent maternal age; parity; vaccination station in rural/urban environment; birth municipality; maternal work; To be born in Baby-Friendly Hospital; birth weight; sex of the baby; hospitalization because of diseases
Caminha et al^6 ^(2010)	Pernambuco, 2006	1,568 babies < 6 m/ PESN	Hierarchical Poisson Regression	Moderate	8.5% (at 6 m)	EBF at 4 m	Metropolitan area [PR = 1.4] Maternal age (≥ 36 years) < 20 years [PR = 15] 20 to 35 years [PR = 1.6] Maternal education (none) From 1^st^ to 4^th^ grade [PR = 1.2] From 5^th^ to 8^th^ grade [PR = 1.7] 9^th^ grade or more [PR = 1.8] Baby of the female sex [PR = 1.3]	Area (urban/rural); water supply; *per capita* income; number of residents; prenatal information on BF; no. of prenatal visits; childbirth type; birth weight; maternal work; to be attended by FHS
Carvalhaes et al^7^ (2007)	Botucatu, SP, 2004	380 babies < 4 m/AMAMUNIC	Hierarchical logistic regression	Moderate	38.0% (< 4 m)	Interruption of EBF	Difficulty in starting breastfeeding [OR = 1.57] Use of a pacifier [OR = 2.63]	Maternal education; parity; maternal work; maternity leave; financing of the hospital; childbirth type; birth weight
Leone et al^21^ (2012)	Sao Paulo, SP, 2008	724 babies < 6 m/AMAMUNIC	Logistic regression	Moderate	39.6% (< 6 m)	Interruption of EBF	Maternal work [OR = 2.11] Child’s age in days [OR = 1.01] Use of a pacifier [OR = 3.02]	Maternal education; childbirth type; birth in BFH; birth weight; sex of the baby; BF in the 1^st^ hour of life
Martins et al^23 ^(2011)	Feira de Santana, BA, 2004-2005	1,309 babies < 1 m/ household visit	Logistic regression	Moderate	59.3% (at the 1^st^ m)	Interruption of EBF	Maternal education: ≤ high school [OR = 1.35] Primiparity [OR = 1.41] Income < 1 minimum wage [OR = 1.27] Lack of guidance on BF in the hospital [OR = 1.53] Tiredness [OR = 1.18]	Maternal age; childbirth type
Nascimento et al^28^ (2010)	Joinville, SC, 2005	1,470 babies < 6 m/ AMAMUNIC	Poisson Regression	Moderate	71.2% (1^st^ month) 61.0% (2^nd^ month) 46.3% (3^rd^ month) 44.2% (4^th^ month) 31.3% (5^th^ month) 13.3% (6^th^ month)	Interruption of EBF	Maternal education < 12 years [PR = 1.59] Child’s age ≥ 90 days [PR = 1.53] Use of a pacifier [PR = 1.69]	Maternal age; parity; birth in BFH; childbirth type; sex of the baby; birth weight; primary care financing; professional who took care of the child; maternal work
Neves et al^29^ (2014)	255 municipalities of N and NE regions, 2010	9,060 babies < 6 m/ Chamada Neonatal	Poisson Regression	Moderate	39.9% (< 6 m)	EBF	Place of residence: capital [PR = 1.17] Maternal age (< 20 years) 20-34 years [PR = 1.14] ≥ 35 years [PR = 1.28] Child’s age (0 month) 1 month [PR = 0.77] 2 months [PR = 0.68] 3 months [PR = 0.53] 4 months [PR = 0.37] 5 months [PR = 0.16] BF in the 1^st^ hour of life [PR = 1.16	Maternal education; skin color; prenatal care; prenatal financing; prenatal guidance on BF; childbirth type; maternity financing; joint accommodation; sex of the baby; birth weight; recent visit of medical agent
Parizoto et al^30^ (2009)	Bauru, SP, 2006	509 babies < 6 m/AMAMUNIC	Logistic regression	Moderate	24.2% (< 6 m) 3.9% (at 6 m)	Interruption of EBF	Use of a pacifier [OR = 2.03]	Maternal age; maternal education; parity; childbirth type; maternity financing; birth weight; maternal work
Pereira et al^31 ^(2010)	Rio de Janeiro, RJ, 2007	1,029 babies < 6 m/patients of 27 basic units.	Hierarchical Poisson Regression	Moderate	58.1% (< 6 m)	EBF	White skin color [PR = 1.20] Living with the partner [PR = 1.72] Previous breastfeeding [PR = 1.27] Prenatal information on BF [PR = 1.27] EBF at discharge [PR = 2.01] Guidance on BF in group [PR = 1.14] Guidance on how to position the baby to suck [PR = 1.20] Baby’s age in months [PR = 0.83]	Maternal age; parity; no. of goods in the residence; no. of prenatal visits; birth in Baby-Friendly Hospital (or in the process of accreditation); childbirth type; birth weight; sex of the baby; maternal work; primary care unit type; guidance on free demand, milk expression, EBF period, and nonuse of bottle; maternal satisfaction with the support received in the primary care unit to breastfeed;
Queluz et al^34 ^(2012)	Serrana, SP, 2009	275 babies < 6 m/AMAMUNIC	Logistic regression	Moderate	29.8% (< 6 m)	Interruption of EBF	Maternal work outside the home without maternity leave [OR = 3.08] Does not work outside the home [OR = 2.26]	Maternal age; parity; maternal education; birth in Baby-Friendly Hospital; childbirth type; birth weight; use of a pacifier; childcare financing
Rito et al^38 ^(2013)	Rio de Janeiro, RJ, 2007-2008	4,092 babies < 6 m/patients of 56 primary care units.	Poisson Regression	Moderate	47.6% (< 6 m)	EBF	Performance in the Breastfeeding-Friendly Primary Care Unit Initiative: superior [PR = 1.34] intermediate [PR = 1.17] Primary Care unit [PR = 1.10] No maternal work [OR = 1.75] Child’s age in days [PR = 0.99]	To have received guidance/help from the hospital to breastfeed
Vannuchi et al^44^ (2005)	Londrina, PR, 2002	988 babies < 6 m/AMAMUNIC	Logistic regression	Moderate	21.0% (< 6 m)	Interruption of EBF	Primiparity [OR = 1.63] Use of a pacifier [OR = 2.23] Outpatient monitoring in public network [OR = 2.08]	Birth weight; baby’s age in days
Venâncio et al^46^ (2002)	84 municipalities in the State of Sao Paulo, 1998	11,481 babies < 6 m/AMAMUNIC	Logistic regression	Moderate	From 0% (in 10 cities) to 54.0% (in 1 city)	Interruption of EBF	Adolescent maternal age [OR = 1.20] Maternal age (≥ 13 years) 9-12 years [OR = 1.54] 5-8 years [OR = 1.94] Until 4 years [OR = 2.28] Primiparity [OR = 1.27] Birth in Baby-Friendly Hospital [OR = 1.49] Municipality with Baby-Friendly Hospital [OR = 2.28]	Maternal work
Venâncio et al^47^ (2006)	111 municipalities in the State of Sao Paulo, 1999	34,345 babies < 6 m/AMA	Multilevel Logistic Regression	Moderate	13.9% (< 6 m) 4.0% (at 6 m)	EBF	Maternal age (< elementary school) elementary school [OR = 1.15] some high school [OR = 1.18] high school [OR = 1.58] ≥ some college [OR = 1.91] Maternal age (from 11 to 17 years) 18-19 years [OR = 1.17] 20-24 years [OR = 1.43] 25-29 years [OR = 1.52] 30-34 years [OR = 1.52] ≥ 35 years [OR = 1.22] Multiparity [OR = 1.41] Birth weight (< 1.500 g) 2,000-2,499 g [OR = 1.29] 2,500-2,999 g [OR = 1.52] ≥ 3,000 g [OR = 1.73] Baby of the female sex [OR = 1.12] Childcare private financing [OR = 1.10] Municipalities with 4 to 5 pro-BF actions [OR = 1.53]	Municipality of residence; population size; human development index (SES/SP); childbirth type; Birth in Baby-Friendly Hospital; maternal work
Audi et al^2^ (2003)	Itapira, SP, 1999	346 babies < 6 m/AMAMUNIC	Logistic regression	Weak	64.8% (at the 1^st^ m) 9.6% (between the 4^th^ and the 6^th^ month)	Interruption of EBF	Childbirth type (caesarean) [OR = 1.78] Use of a pacifier (yes) [OR = 4.41]	Place of residence; maternal age; maternal education; parity; financing of the hospital; birth weight; maternal work; maternity leave; childcare financing
Damião^10^ (2008)	Rio de Janeiro, RJ, 1998 and 2000	2,459 babies < 4 m/AMAMUNIC	Logistic regression	Weak	22.7% (< 4 m)	EBF	Maternal age in years [OR = 1.02] Maternal education college degree [OR = 1.93] Maternal work [OR = 0.59] child’s age in days [OR = 0.99]	Parity; birth in a BFH; birth weight
Fernandes et al^15^ (2012)	Rio de Janeiro, RJ, 2005-2008	592 babies < 1 m/ patient of 4 basic units	Logistic regression	Weak	About 75.0% (< 1 m)	Interruption of EBF	Pregestational obesity [OR = 2.14] Overweight with excessive gestational weight gain (GWG) [OR = 2.29] Obesity with insufficient GWG [OR = 3.10] Obesity with excessive GWG [OR = 3.33] Excessive GWG [OR = 1.50]	Maternal education; smoking in pregnancy; no. of prenatal visits; childbirth type; birth weight; social support; social network
França et al^16^ (2007)	Cuiaba, MS, 2004	275 babies < 6 m/AMAMUNIC	Logistic regression	Weak	34.5% (< 6 m)	Interruption of EBF	Maternal age (≥ 35 years) < 20 years [OR = 3.54] from 20 to 34 years [OR = 3.13] Maternal education Elementary and high school [OR = 2.31] Primiparity [OR = 2.20] Use of a pacifier [OR = 3.26]	Birth in a hospital with Milk Bank; childbirth type; maternal work; primary care financing
Gusmão et al^18^ (2013)	Porto Alegre, RS, 2009	341 babies < 6 m children of adolescent mothers/Household survey	Hierarchical Poisson Regression	Weak	37.8% (< 6 m)	EBF	Maternal education: high school [PR = 1.53] Multiparity [PR = 1.57] Child’s age in months [PR = 0.76]	Maternal age; Skin color; Grandmother’s education; Attends school; Marital status; Owns an income; Social class; Prenatal financing; No. of prenatal visits; Birth in BFH; childbirth type; Desire for pregnancy; Partner’s attitude; Family reaction; Emotional indicators; Expectation regarding the future; Self-worth; Psychological distress; Birth weight; 5^th^ minute Apgar; Sex of the baby; Caregiver; Degree of difficulty in taking care of the baby; Perception of the baby’s health
Vieira et al^51^ (2010)	Feira de Santana, BA, 2004-2005.	1,309 babies with one month of life/interviews	Logistic regression	Weak	59.3% (at the end of the 1^st^ month)	Interruption of EBF	Lack of prior experience with breastfeeding [OR = 1.24] Pre-set schedule to breastfeed [OR = 1.42] Use of a pacifier [OR = 1.53] Presence of nipple fissures [OR = 1.25]	Birth weight; BF in the first hour of life

EBF: Exclusive Breastfeeding; FHS: Family Health Strategy; BF: breastfeeding

**Table 2:** Included sectional studies on factors associated with exclusive breastfeeding.

Author/Publishing year	Local/year of implementation	Sample (n)/data source	Statistical analysis	Quality Score	EBF Prevalence (age group)	Outcome	Factors associated with EBF	Factors evaluated without statistical significance
Chaves et al^9 ^(2007)	Hospital Municipal de Itauna, MG, 2003	238 babies monitored until 6 m	Cox Regression	Strong	62.6% (1 month); 19.5% (4 months); 5.3% (6 months)	Interruption of EBF	Intention to breastfeed (> 24 months): < 12 months [RR = 1.67] from 12 to 23 months [RR = 1.74] Birth weight > 2,500 g [RR = 1.92] Use of a pacifier [RR = 1.49]	Basic sanitation; skin color; maternal education; marital status; parity; income; information on the BF technique; use of alcohol or tobacco; health insurance; no. of prenatal visits; gestational age; sex of the baby; maternal work; maternity leave; family support; time until the first breastfeed; complications after childbirth
Demétrio et al^11^ (2012)	Laje and Mutuipe, BA, 2005-2008	531 babies monitored until 6 m/AMACOMP	Cox Regression	Strong	74.7 days (median)	Interruption of EBF	Urban residence [HR = 1.61] No prenatal care [HR = 2.73]	Housing condition; maternal age; skin color; maternal education; maternal stature; anthropometric nutritional status; childbirth type; birth weight; gestational age; sex of the baby; maternal work
Mascarenhas et al^24^ (2006)	Pelotas, RS, 2002-2003	940 babies monitored until 3 m	Hierarchical logistic regression	Strong	39,0% (at 3 months)	Interruption of EBF before 3 m	Household income (> 6 Minimum Wages - MW): from 1.1 to 3 MW [OR = 1.60] Paternal education (≥ 9 years): from 0 to 4 years [OR = 1.61] Maternal work [OR = 1.76] Use of a pacifier [OR = 4.25]	Skin color; maternal age; maternal education; parity; no. of prenatal visits; smoking in pregnancy; birth weight; gestational age; sex of the baby
Santo et al^40^ (2007)	Hospital das Clínicas (Porto Alegre, RS), 2003	220 babies with up to 6 m (with birth weight ≥ 2,500 g)	Cox Regression	Strong	54.0% (at the 1st m) 6.6% (at 6 m	Interruption of EBF	Adolescent maternal age [HR = 1.48] < 6 prenatal visits (< 6 visits) [HR = 1.60] Use of a pacifier [HR = 1.53] No. of negative evaluations of latching at maternity [HR = 1.29]	Skin color; maternal education; parity; marital status; duration of the BF of the prior child; living with the grandmother of the child; prenatal information on BF; participation in a group of pregnant women in prenatal care; postpartum mammillary lesion; no. of negative evaluations of positioning in the breast at maternity
Silva et al^43^ (2008)	Pelotas, RS, 2002-2003	951 babies monitored until one month of age.	Hierarchical logistic regression	Strong	60.0% (at the first month of life)	Interruption of EBF in the 1^st^ month of life	Paternal education (≥ 9 years): from 5 to 8 years [OR = 1.31] from 0 to 4 years [OR = 1.63] Paternal age (greater than 35 years) from 20 to 34 years [OR = 1.45] less than 20 years [OR = 1.43] Use of a pacifier [OR = 2.45]	Skin color; no. of visits in the prenatal care; smoking in pregnancy; birth in Baby-Friendly Hospital; birth weight; sex of the baby; maternal work
Vieira et al^52 ^(2014)	Feira de Santana, BA, 2004-2005	1,344 children monitored until 6 m of age	Hierarchical Cox Regression	Strong	89 days (median)	Interruption of EBF	Maternal education ≤ 8 years [HR = 1.34] < 6 prenatal visits [HR = 1.48] Prenatal public financing [HR = 1.34] Birth in Baby-Friendly Hospital [HR = 0.85] Guidance on BF in the hospital [HR = 0.80] Partner favorable to breastfeeding [HR = 0.62] Maternal work [HR = 1.73] The mother limits breastfeeding at night [HR = 1.58] Nipple fissure [HR = 2.54]	Skin color; maternal age; parity; prior experience with BF; the mother lives with the father of the child; the mother participated in a prenatal group on BF; childbirth type; BF in the first hour of life; sex of the baby
Bueno et al^5^ (2003)	University Hospital in Sao Paulo, SP, 1998-1999	383 babies monitored until 6 m	Cox Regression	Weak	23 days (median); 38.5% (1 m); 13.8% (3 m); 1.6% (6 m).	Interruption of EBF	Maternal age ≤ 25 years [HR = 1.22] Maternal education (college degree): Elementary school [HR = 2.13] High school [HR = 1.78] Baby of the female sex [RR = 1.22]	To live in a slum; skin color; parity; marital status; number of consumer goods; number of prenatal visits; smoking habit; childbirth type; birth weight

BF: breastfeeding; EBF: exclusive breastfeeding

Most of the studies were conducted in towns and covered 77,866 children. Regarding Brazilian regions, 14 studies were carried out in the southeastern, six in the south, five in the Northeast, one in the Midwest, and one in the Northern region. The systematic review included studies conducted between 1998 and 2010 (Tables 1 and 2).

The punctual prevalence of exclusive breastfeeding at six months ranged from 3.9% in Bauru,^30^ SP, Southeastern Brazil, to 8.5% in Pernambuco, Northeastern Brazil, both in 2006.^6^ Regarding the WHO indicator, the prevalence of exclusive breastfeeding in children younger than six months of life, resulting from the survey of this population, ranged from 0% in 10 cities in the state of Sao Paulo, Southeastern Brazil, in 1998^46^ to 58.1% in the city of Rio de Janeiro, Southeastern Brazil, in 2007^31^ (Tables 1 and 2).

Factors associated with exclusive breastfeeding were organized into hierarchical levels (Table 3), being the following the most often exploited (more than a fifth of the 27 studies): place of residence, skin color, maternal age and education, parity, marital status, number of prenatal visits, birth in Baby-Friendly Hospital (BFH), childbirth type, birth weight, sex of the newborn, maternal work, age of the child, financing of the primary health care of the child unit, and the use of a pacifier.

**Table 3:** Factors investigated regarding the association with exclusive breastfeeding, organized by hierarchical level, frequency of use, and number of times they were associated with exclusive breastfeeding in a statistically significant way.

Factor and level	Studies	Association	Factor and level	Studies	Association	Factor and level	Studies	Association	Factor and level	Studies	Association

Contextual	n	N	Distal intermediate	N	n	Proximal intermediate	n	n	Proximal	n	n
Total of actions in breastfeeding	1	1	Anthropometric nutritional status	2	1	Birth in Baby-Friendly Hospital	12	1	Maternal work	20	6
Population size	1	0	Desire for pregnancy	1	0	Birth in a hospital with Human Milk Bank	1	0	Maternity leave	4	1
Compound population indexes	2	0	Smoking	4	0	Type of financing of the maternity or hospital	4	0	Tiredness and emotional indicators	2	1
Place of residence	7	3	Alcoholism	1	0	Guidance on breastfeeding in the maternity or professional service	4	2	Knowledge about breastfeeding technique	1	0
Basic sanitation	3	0	Prenatal visits	12	3	Number of negative evaluations of latching	1	1	Difficulties in breastfeeding	3	2
Distal			Information on breastfeeding in prenatal care	4	1	Number of negative evaluations of breastfeeding position	1	0	Family support	3	1
									Baby’s caregiver	1	0
Skin color or race	10	1	Participation in a prenatal group	2	0	Rooming-in	1	0	Living with the child’s grandmother	1	0
Maternal age	20	8	Prenatal financing	4	1	Intention to breastfeed	1	1	Child’s age	8	8
Maternal education	23	11				Childbirth type	19	2	Child’s health	2	0
Parity	19	6				Time until the first breastfeed	5	2	Use of a pacifier	16	15
Prior experience with breastfeeding	4	2				Exclusive breastfeeding at discharge	1	1	Type of financing of primary health care	6	2
Marital status	6	1				Birth weight	21	3	Type of primary care unit	4	1
Paternal age	1	1				Gestational age	4	0	Guidance on breastfeeding in group	1	1
Paternal or grandmother’s education	3	2				Immediate postpartum complications and Apgar	2	0	Guidance on latching or positioning	1	1
Household income	5	2				Sex of the newborn	14	3	Other guidelines on breastfeeding	1	0
Number of people in the house	1	0							Mother’s satisfaction regarding the support received	1	0
Number of goods in the residence	2	0							Assistance by a Breastfeeding-Friendly Primary Care Unit	2	2

The factors most frequently associated with exclusive breastfeeding (factors investigated in at least six studies and which have showed association in at least one-third of the studies in which they were investigated) were (according to the category positively associated with the outcome): place of residence (residence in the capital, in the metropolitan area, or in rural areas), intermediate maternal age, maternal education, lack of maternal work, age of the child (descending), the nonuse of a pacifier, and financing of primary health care (private) (Tables 1, 2, and 3).

The studies listed, in total, 36 factors associated with exclusive breastfeeding, 11 classified as distal, four as distal intermediate, nine as proximal intermediate, and 12 as proximal (Table 3).

Of the selected studies, eight used hierarchial theoretical model to identify factors associated with exclusive breastfeeding before starting the statistical modeling, and only one considered contextual variables^47^ (Tables 1 and 2).

**Figure 2:**
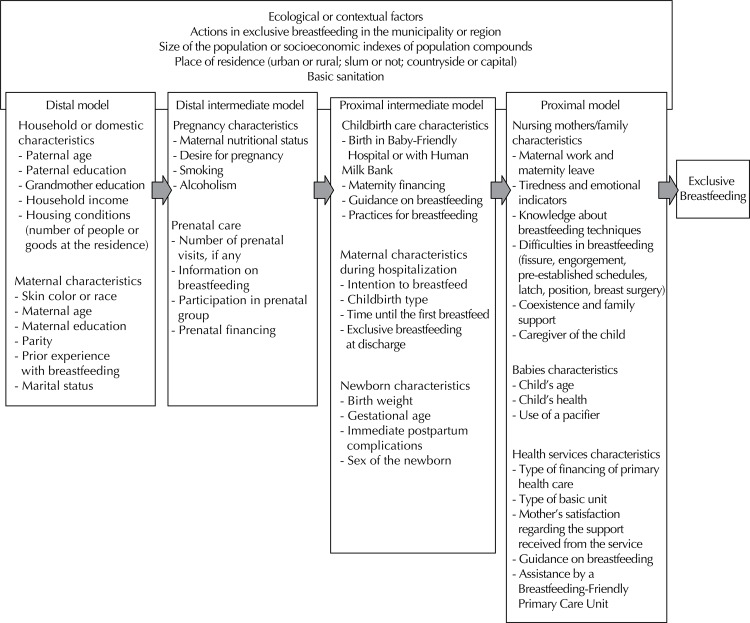
Hierarchical theoretical model of factors associated with exclusive breastfeeding.

Based on the factors listed in the analysis of the 27 selected studies, was created a hierarchial theoretical model of the factors associated with exclusive breastfeeding. Some were constituted of the group of similar factors, such as “difficulties in breastfeeding”, which grouped the following variables: nipple fissure, pre-set schedule to breastfeed, and difficulties in latching or positioning. Similarly, were grouped as “emotional indicators” the variables: maternal self-worth and psychological distress (Figure 2).

## DISCUSSION

A systematic review of Brazilian epidemiologic studies showed a relevant production of studies from the late 1990s having exclusive breastfeeding as outcome, which were conducted mainly in the Southeast region of Brazil. Most of the studies selected for this review showed moderate quality, and only a quarter of the articles had a longitudinal design. Therefore, the evidence of the factors associated with exclusive breastfeeding in children under six months of age in Brazil found in this review can be considered as moderate.

The number of variables listed in epidemiological studies, and used to explain the duration of exclusive breastfeeding, was high, and the discussion of the findings of this systematic review was marked by the organization of the variables in hierarchical levels. Because of the diversity of backgrounds and factors investigated, the use of summary measures of association derived from meta-analysis techniques was considered invalid.

Among distal factors, the place of residence was the contextual variable investigated the most, and the results were discordant, and sometimes the urban environment,^29^ sometimes the rural^11^ were associated with exclusive breastfeeding. Most of distal factors seem to represent maternal socioeconomic factors. Maternal education was the factor most widely investigated, with almost half of the studies having observed an association between maternal education and exclusive breastfeeding, and the findings were unanimous: the low education level was associated with the interruption of exclusive breastfeeding. In epidemiological studies, the socioeconomic gradient is reproduced, in general, in a health gradient.^22^ The national research on breastfeeding also report these differences, in which mothers with higher education exclusively breastfeed for more time.^d^

The variable “skin color or race”, in turn, may represent customs, social norms and traditions,^27^ besides income^8^ and social relations.^e^ Considering the national surveys, white mothers breastfed exclusively for more time,^b^ but only one study found an association between skin color and higher prevalence of exclusive breastfeeding.^31^

Maternal age and parity may represent the experience with breastfeeding.^31,51^ All studies that investigated them observed an association between greater parity and exclusive breastfeeding.^16,18,23,44,46,47^ Regarding maternal age, the intermediate ages seem to be protective for exclusive breastfeeding, because both teenage mothers^5,29,40,46,47^ and those with 35 years of age or more^6,10,16^ interrupt it prematurely.

Considering the distal intermediate factors concerning pregnancy, the number of prenatal visits was the variable most frequently investigated. The three studies that found an association between this variable and the outcome indicated the low number of prenatal visits as a risk factor for exclusive breastfeeding. Santo et al^40^ and Vieira et al^52^ consider that low compliance to prenatal care may represent women who are less careful with their health; on the other hand, Demétrio et al^11^ consider that this low compliance may reflect low access to sources of information on breastfeeding.

The public or private service pervades all hierarchical levels evaluated: prenatal care (distal intermediate level), childbirth (proximal intermediate), and childcare (proximal). The private primary care was associated with the outcome in three of 10 studies.^44,47,52^ This variable may represent both the access to health services and the maternal socioeconomic status, for the access to health services may be determined by more distal variables such as skin color, sex, education, and income.^36^

Among the proximal intermediate factors, the birth weight was the one most widely used, finding a positive association between children with adequate birth weight and exclusive breastfeeding in three of the 21 studies that investigated it.^9,44,47^ This may be explained because children with low birth weight are more likely to spend more time hospitalized in neonatal unit, thus spending more time separated from their mothers.^41^ In addition, these children may have more difficulties in initiating or maintaining breastfeeding, since both the frequency and the pressure of the suction increase as the gestational age and weight of newborn increase.^25^

Gestational age, in turn, was an indicator hardly used in the studies, for differences or biases in the rankings of this variable that may occur.^42^ Although no studies have found an association between this variable and the outcome, it is suggested to maintain it in the studies.

Another factor widely used in the studies was the childbirth type, however, only two studies have found an association between the vaginal childbirth and higher prevalence of exclusive breastfeeding.^1,2^ The vaginal childbirth contributes to the timely initiation of breastfeeding,^3^ being possible to assume that it can also provide its maintenance in exclusive mode. Another hypothesis would be the possible relationship between socioeconomic characteristics and access to public health services,^17^ since both the vaginal childbirth^13^ and timely initiation of breastfeeding are more practiced at these services.^3^

Among the proximal intermediate factors studied, those that assess the guidelines received in hospital^23,52^ (positive association with the outcome), the difficulties to breastfeed during hospitalization^7^ (negative association), and exclusive breastfeeding on discharge^31^ (positive association) may be the most adequate to evaluate peri-partum related aspects which may determine the duration of exclusive breastfeeding.

The variable “sex of the baby” was used in 14 studies, considering that two^6,47^ found a positive association between female and one between male^5^ and exclusive breastfeeding. The prevalence of breastfeeding among girls was higher in the capitals of the entire Brazil;^b^ however, it is unclear whether this increased prevalence is due to some cultural aspect such as the belief that boys need greater nutritional intake by other foods in addition to breast milk.^32,47^

Regarding the proximal factors considered, the use of a pacifier was the factor most strongly associated with the interruption of exclusive breastfeeding.^2,7,9,16,21,24,28,30,43,44,51,52^ The use of a pacifier may lead to the reduction in breastfeeding frequency, interfering in breast demand, and possibly changing the baby’s oral dynamics.^50^ A Brazilian study concluded that, in addition to the causal relationship between the use of a pacifier and breastfeeding interruption be unclear (it is unknown whether the use of a pacifier is a marker of the interruption of breastfeeding, or if it is a cause of the same), the process of using pacifiers is dynamic, with children starting or stopping the use of a pacifier throughout the period.^50^ In a randomized study conducted in Canada,^19^ the authors observed that the use of a pacifier can be a marker of the interruption of breastfeeding or of low motivation to breastfeed rather than be the cause of the interruption of breastfeeding.

Maternal work was a variable widely used in studies,^1,2,6,7,9-11,16,21,24,28,30,31,34,38,43-47,52^ and in the six studies that found statistically significant association, it showed a negative association with the outcome. However, this variable must be investigated considering if the mother is or is not on maternity leave.^51^ Mothers that work outside the home with maternity leave would have better conditions to maintain exclusive breastfeeding during the maternity leave period.

Most of the studies based on surveys did not consider the child’s age, but the probability to be exclusively breastfed decreases as the age of the child increases. All the studies that used this variable found an association between the descending age (or early age) of the child and exclusive breastfeeding.^1,10,18,21,28,29,31,44^

Among all the variables considered proximal, those that evaluate the access to information or guidance on breastfeeding that women receive in primary health care services could be those more directly associated with exclusive breastfeeding. However, only Pereira et al^31^ used this variable, noting that guidelines in group and on positioning and latching of the baby were associated with a higher prevalence of exclusive breastfeeding.

Evaluating statistical modeling strategies, less than a quarter of the studies included in this review adopted a theoretical model prior to analysis, organizing the variables in hierarchical levels.^6,7,18,24,31,43,52^ Although expendable, to create this conceptual model is important because it requires prior knowledge about the social and biological factors associated with outcome, assisting to establish an order of logic input variables in the model based on hierarchy of factors and not considering only purely statistical criteria.^49^ Thus, a hierarchical theoretical model was proposed, including factors identified in the studies of this systematic review, which can assist in planning data collection and statistical modeling strategy of epidemiological studies related to exclusive breastfeeding.

Public policies to promote, protect, and support breastfeeding adopted in Brazil since the 1980s have contributed to the increase in the median duration of breastfeeding and its exclusive mode across the Country.^35^ These policies, however, cannot be considered as an individual attribute: having a childbirth in Baby-Friendly Hospital or maternity with Human Milk Bank may depend on the context in which the woman lives as well as her access to these services.

In addition, the local contexts within each city (districts, neighborhoods, surroundings) may vary: in the city of Rio de Janeiro, e.g., a great variation in the adoption of the 10 steps to successful breastfeeding (recommended by the Breastfeeding-Friendly Primary Care Unit Initiative) are found between the units of primary health network.^37^ In addition, disparities between public and private health units must be considered, such as those observed in the adoption of breastfeeding in the first hour of life in hospitals (recommended by the Baby-Friendly Hospital Initiative).^3^

Considering this possible context effect, it is plausible that nursing mothers who are residing in the same regions or municipalities (including districts, neighborhoods, or census units) share social and economic factors (contextual factors) that influence the duration of exclusive breastfeeding, e.g., standards and attitudes toward breastfeeding; the organization and access to primary health services in their neighborhood; and the level of action and policies for promoting, protecting, and supporting breastfeeding. In fact, variables such as socioeconomic indexes and the number of pro-breastfeeding actions existent in certain regions have already been used for the evaluation of factors associated with exclusive breastfeeding.^47^

Many factors used in analytical epidemiological studies and its directionality in association with exclusive breastfeeding were identified and described, noting the frequency with which they are used and the heterogeneity of categories and cutoff points. Instead of defining the effect of each of the factors identified in the systematic review by meta-analysis, we decided to discuss them according to a hierarchical theoretical model.

Some recommendations concerning the findings of this study include the completion of further studies in the North and Midwest of the Country, as well as the encouragement to academic work on little explored factors in association with exclusive breastfeeding.

The use of a conceptual theoretical model prior to statistical analysis, preferring the hierarchical organization of variables in relation to the proximity to the outcome, may help the choice of variables to be included in the studies and to evaluate the intermediation of more proximal variable blocks in relation to the more distal ones.

It is suggested that future studies consider context variables to investigate the association with exclusive breastfeeding, since inclusion of contextual variables with concomitant multilevel models is a useful strategy for the adequacy of these models.^12^ Studies covering the triangulation of qualitative and quantitative methods^26^ to the understanding of the relation of some factors with exclusive breastfeeding could also contribute to a better understanding of the subject.

The main limitation of this systematic review was the selection bias, since abstracts published in conference proceedings were not included, which is called the “grey literature”.^33^ Another limitation is the possibility that relevant studies have not been found by the search strategy used. The possible subjectivity of the authors in the evaluation and selection of articles was minimized by the independent search of the literature, by the standardized form-filling, and by assessing the quality of the articles selected for the review.

In conclusion, the study of determinants of exclusive breastfeeding is of vital importance for public health, and epidemiological studies have an important role for the understanding of this theme in Brazil. However, the emergence of new and more sophisticated statistical tools, as well as the growing complexity of explanatory models and the context effects of the factors associated with exclusive breastfeeding, brings a new challenge to scholars of the topic: the careful use of these resources and the dissemination of the results in a clear and purposeful way, directed to the development and improvement of public policies for promoting, protecting, and supporting breastfeeding which are reflected in the health and well-being of the population.

## Footnotes

a Ministério da Saúde, Secretaria de Atenção à Saúde, Departamento de Ações Programáticas e Estratégicas. II Pesquisa de prevalência de aleitamento materno nas capitais brasileiras e Distrito Federal. Brasília (DF): Ministério da Saúde; 2009. (Série C. Projetos, programas e relatórios).

b Ministério da Saúde. PNDS 2006: pesquisa nacional de demografia e saúde da criança e da mulher. Brasília (DF): Ministério da Saúde; 2008.

c World Health Organization. Indicators for assessing infant and young child feeding practices. Part 1: definitions. Conclusions of a consensus meeting held 6-8 November 2007 in Washington, DC, USA. Geneva: World Health Organization; 2007.
