# Supplementary material for: Factors associated with exclusive breastfeeding in the first six months of life in Brazil: a systematic review
Source: Rev Saude Publica. 2015 Dec 16;49:91. doi: 10.1590/S0034-8910.2015049005971 (PMC4687824; doi:10.1590/S0034-8910.2015049005971)
Supplement: Translation

# Translation: Fatores associados ao aleitamento materno exclusivo nos primeiros seis meses de vida no Brasil: revisão sistemática

**Authors:** Cristiano Siqueira Boccolini, Márcia Lazaro de Carvalho, Maria Inês Couto de Oliveira

**Affiliations:** I Laboratório de Informação em Saúde. Instituto de Comunicação e Informação Científica e Tecnológica em Saúde. Fundação Oswaldo Cruz. Rio de Janeiro, RJ, Brasil; II Departamento de Epidemiologia e Métodos Quantitativos em Saúde. Escola Nacional de Saúde Pública. Fundação Oswaldo Cruz. Rio de Janeiro, RJ, Brasil; III Departamento de Epidemiologia e Bioestatística. Instituto de Saúde Coletiva. Universidade Federal Fluminense. Niterói, RJ, Brasil

**Keywords:** Aleitamento Materno, Comportamento Materno, Fatores de Risco, Fatores Socioeconômicos, Revisão

## Abstract

**OBJETIVO:**

Identificar fatores associados ao aleitamento materno exclusivo nos primeiros seis meses de vida no Brasil.

**MÉTODOS:**

Revisão sistemática de estudos epidemiológicos conduzidos no Brasil com o aleitamento materno exclusivo como desfecho. Foram utilizadas as bases de dados Medline e Lilacs. Após a seleção de artigos, foi proposto um modelo teórico hierarquizado, segundo a proximidade da variável com o desfecho.

**RESULTADOS:**

Dos 67 artigos identificados, foram selecionados 20 estudos transversais e sete de coorte, conduzidos entre 1998 e 2010, compreendendo 77.866 crianças. Foram identificados 36 fatores associados ao aleitamento materno exclusivo, sendo mais frequentemente associados os fatores distais: local de residência, idade e escolaridade maternas, e os fatores proximais: trabalho materno, idade da criança, uso de chupeta e financiamento da atenção primária em saúde.

**CONCLUSÕES:**

O modelo teórico desenvolvido pode contribuir para a condução de futuras pesquisas e os fatores associados ao aleitamento materno exclusivo podem subsidiar políticas públicas em saúde e nutrição.

## INTRODUÇÃO

O aleitamento materno é um tema crucial para a saúde pública, pois afeta diretamente os padrões de saúde e de mortalidade das populações.^4,14,20,39,53^ A prevalência e a duração do aleitamento materno parcial ou exclusivo aumentaram em todas as camadas sociais e regiões do Brasil entre as décadas de 1990 e 2010.^35,45,a,b^ Parte dessa tendência pode ser atribuída às políticas nacionais de promoção, proteção e apoio ao aleitamento materno.^35^

Os diferentes contextos sociais e culturais podem influenciar a prática do aleitamento materno exclusivo e seus determinantes. Estudo conduzido em cidades de três países observou que maiores níveis de escolaridade materna estavam relacionados tanto com maiores prevalências de aleitamento materno exclusivo em Santos, SP, Brasil, quanto com menores prevalências na Cidade do México, México, e em Sula e Tegucigalpa, Honduras.^32^

Buscando maior homogeneidade populacional, esta revisão restringiu-se ao contexto brasileiro, já que os determinantes do aleitamento materno exclusivo podem se comportar diferentemente em culturas diversas.

O objetivo deste estudo foi identificar os fatores associados ao aleitamento materno exclusivo nos primeiros seis meses de vida no Brasil.

## MÉTODOS

Foram analisadas publicações de estudos epidemiológicos conduzidos no Brasil sobre fatores associados ao aleitamento materno exclusivo. Foi realizada pesquisa bibliográfica nas bases Medline (via PubMed) e Lilacs. Não houve delimitação por período nem por idioma. Foi feita busca manual das referências incluídas na bibliografia de cada artigo.

As buscas foram conduzidas em julho de 2014, de forma independente, por dois revisores. Os termos de busca avançada para o PubMed foram: (exclusive[All Fields] AND (“breast feeding”[MeSH Terms] OR (“breast”[All Fields] AND “feeding”[All Fields]) OR “breast feeding”[All Fields] OR “breastfeeding”[All Fields]) AND (“Brazil”[MeSH Terms] OR “Brazil”[All Fields])) AND (determinants[All Fields] OR factors[All Fields] OR (“epidemiology”[Subheading] OR “epidemiology”[All Fields] OR “epidemiology”[MeSH Terms]))”.

Na base de dados Lilacs, foram pesquisados, no modo “detalhes da pesquisa”, os seguintes termos: “tw:(tw:(exclusive AND breastfeeding (epidemiology OR determinants OR factors)) AND (instance:“regional”) AND (db:(“LILACS”))) AND (instance:“regional”) AND (mj:(“Aleitamento Materno”))”. Também foram utilizados os termos em português: “tw:(aleitamento AND materno AND exclusivo) AND (instance:“regional”) AND (db:(“LILACS”) AND mj:(“Aleitamento Materno”)) OR fatores)) AND (instance:“regional”) AND (db:(“LILACS”))”. O termo “Brasil” não foi empregado nessa base de dados, pois esta possui somente artigos publicados em periódicos da América Latina e Caribe.

Foram incluídos estudos epidemiológicos observacionais analíticos em que o aleitamento materno exclusivo era tratado como desfecho, com ajuste dos fatores estudados entre si e por possíveis fatores de confundimento, que adotaram a definição da World Health Organization (WHO)^c^ para aleitamento materno exclusivo (a criança recebe somente leite humano, diretamente de sua mãe ou extraído, e não recebe mais nenhum outro líquido ou sólido, exceto gotas ou xaropes de vitaminas, suplementos vitamínicos ou medicamentos) e cujo processo de amostragem tenha gerado população representativa de lactentes de maternidades, cidades, estados, ou da nação brasileira.

Nos artigos em que mais de uma faixa etária foi avaliada (mais de um modelo estatístico para avaliar duas ou mais faixas etárias diferentes), foi escolhida a faixa etária de maior idade (com o limite de seis meses), uma vez que o objetivo foi avaliar o desfecho mais próximo à recomendação da WHO^c^ de amamentação exclusiva até os seis meses de idade.

Foram excluídos estudos: com resultados sujeitos a viés de seleção (como perdas superiores a 20,0%) ou com possível viés de informação (como entrevistas realizadas com mães de crianças com mais de um ano de vida); que apresentaram somente o valor de p (sem apresentar as medidas de associação), que consideraram somente a população de nascidos com baixo peso; e revisões bibliográficas (sistemáticas ou não).

No caso de estudos que utilizaram a mesma base de dados e que foram publicados em mais de um artigo (periódicos e anos distintos), foram incluídos os que utilizaram faixas etárias diferentes ou variáveis e métodos analíticos distintos.

Os artigos selecionados foram armazenados sob o *Portable Document Format *(pdf) em diretório compartilhado em nuvem, discriminados pelas bases de dados de obtenção (Medline e Lilacs), e classificados em pastas distintas entre incluídos e excluídos.

A avaliação da qualidade metodológica dos estudos selecionados foi obtida pela adaptação da escala “*Effective Public Health Practice Project: Quality Assessment Tool for Quantitative Studies – QATQS*” (http://www.ephpp.ca/tools.html). Desta escala foram avaliados cinco quesitos (classificados em “fortes”, “moderados” ou “fracos”): 1) viés de seleção; 2) desenho de estudo; 3) fatores de confundimento; 4) métodos de coleta de dados; e 5) tipo de análise empregada para o desfecho. As questões de cegamento (*blinding*) do QATQS não foram utilizadas em nenhum estudo (uma vez que nenhum ensaio clínico foi incluído), e as questões de perda de seguimento não foram aplicadas nos estudos seccionais. No quesito “desenho de estudo”, os estudos seccionais tiveram pontuação inferior aos estudos de coorte, pois nos estudos seccionais a temporalidade entre as variáveis de exposição e o desfecho nem sempre pode ser estabelecida.

Considerando a pontuação final da escala QATQS de cada artigo selecionado, os artigos foram considerados fortes no caso de nenhum dos quesitos ter sido avaliado como fraco; moderado, no caso dos estudos que apresentaram um dos quesitos classificado como fraco; e, fracos, os estudos com um ou mais quesitos assim avaliados.

A extração dos dados foi realizada de forma independente por dois revisores por meio de formulário estruturado, onde foram registrados: último nome do primeiro autor; ano e revista de publicação; local(is) de realização; ano e período de realização; desenho de estudo; população do estudo; plano amostral utilizado; estratégia de seleção dos sujeitos da pesquisa; critérios de inclusão e exclusão; número amostral total; número amostral avaliado; total de perdas e motivo das perdas; faixa etária das crianças estudadas; tipo de desfecho; tipo de análise estatística; fatores de controle ou ajuste do modelo estatístico; resultados do modelo com a medida de associação e significância estatística; prevalência ou mediana de aleitamento materno exclusivo; limitações do estudo; e observações. Em caso de não concordância entre os pares, um terceiro revisor foi consultado.

A tabulação dos dados incluiu: referência do artigo (com o último nome do primeiro autor, revista e ano de publicação); local do estudo e ano da coleta de dados; número amostral avaliado (e fonte de dados); desfecho do estudo (aleitamento materno exclusivo ou sua interrupção); análise estatística empregada; prevalência (ou mediana) do aleitamento materno exclusivo encontrada e a faixa etária dessa prevalência (expressa em meses de vida); fatores associados ao aleitamento materno exclusivo de forma estatisticamente significativa (obtidos dos resultados dos modelos estatísticos), bem como sua medida de associação, e outros fatores avaliados sem associação estatisticamente significativa com o aleitamento materno exclusivo.

Foram construídas duas tabelas, uma para estudos transversais e outra para estudos de coorte. Como os estudos revistos mensuraram a prevalência ou a duração do aleitamento materno exclusivo em diferentes faixas etárias, as tabelas sumárias desse desfecho trazem esta informação.

A etapa seguinte consistiu em analisar individualmente a associação encontrada entre os fatores investigados e o aleitamento materno exclusivo, destacando e quantificando os seguintes aspectos: em quantos estudos esses fatores foram investigados, em quantos foi identificada associação com o aleitamento materno exclusivo nos modelos estatísticos e qual a sua direção.

A última etapa do estudo foi a construção de um modelo teórico hierarquizado (utilizando os pressupostos estabelecidos por Víctora et al)^49^ organizando todos os fatores encontrados de acordo com a proximidade com o desfecho. A seleção dos níveis de alocação das variáveis obedeceu à lógica de classificação cronológica entre fatores presentes antes da gestação, durante a gestação, no pós-parto imediato e do momento da alta hospitalar até os seis meses de vida.

Foram propostos quatro níveis de variáveis, agrupadas em blocos hierarquizados: 1) características distais (contextuais, domiciliares, familiares e maternas); 2) intermediárias distais (da gestação e da atenção pré-natal); 3) intermediárias proximais (da atenção ao parto, características maternas durante a internação hospitalar e características do recém-nascido); 4) proximais (características das nutrizes e da família, dos bebês e dos serviços de saúde).

Para prover parcimônia ao sumário das variáveis identificadas e também para a construção do modelo teórico, a terminologia utilizada em cada artigo para cada variável foi padronizada.

## RESULTADOS

Dos 67 artigos recuperados por busca eletrônica, 44 foram excluídos por não se enquadrarem nos critérios de seleção. Após busca manual, foram incluídos quatro artigos,^21,29,38,44^ totalizando 27 artigos selecionados para análise^5-7,9-11,15,16,18,21,23,24,28-31,34,39,40,43-46,47,51,52^ (Figura 1), dos quais sete são estudos de coorte e 20, estudos seccionais. Destes 20, 12 utilizaram questionários baseados no Projeto Amamentação e Municípios (AMAMUNIC).^48^ Considerando a classificação dos artigos selecionados de acordo com a escala adaptada da QATQS, dos estudos seccionais, 14 foram considerados moderados e seis, fracos (Tabela 1). Entre os estudos de coorte, seis foram considerados fortes e apenas um, fraco (Tabela 2).

**Figura 1:** Fluxograma descritivo das etapas de revisão sistemática nas bases de dados Medline e Lilacs.

**Tabela 1:** Estudos seccionais sobre fatores associados ao aleitamento materno exclusivo.

Autor/Ano de publicação	Local/ano de realização	Amostra (n)/fonte de dados	Análise estatística	Escore de Qualidade	Prevalência AME (faixa etária)	Desfecho	Fatores associados ao desfecho e medida de associação	Fatores avaliados sem significância estatística
Alves et al^1^ (2013)	Barra Mansa, RJ, 2006	707 bebês < 6 m/AMAMUNIC	Regressão Poisson hierarquizada	Moderado	46,7% (< 6 m)	AME	Escolaridade mãe ≤ 8ª série [RP = 0,80] Tipo de parto (cesariano) [RP = 0,84] Idade da criança em dias [RP = 0,99] Uso de chupeta [RP = 0,59] Acompanhamento em Unidade Básica Amiga da Amamentação [RP = 1,19]	Idade materna adolescente; paridade; posto de vacinação no meio rural/urbano; município de nascimento; trabalho materno; nascimento em HAC; peso ao nascer; sexo do bebê; internação hospitalar por patologias
Caminha et al^6^ (2010)	Pernambuco, 2006	1.568 bebês < 6 m/PESN	Regressão Poisson hierarquizada	Moderado	8,5% (aos 6 m)	AME aos 4 m	Região metropolitana [RP = 1,4] Idade materna (≥ 36 anos) < 20 anos [RP = 1,5] 20 a 35 anos [RP = 1,6] Escolaridade materna (nenhuma) 1^a^ a 4^ a^ série [RP = 1,2] 5^ a^ a 8^ a^ série [RP = 1,7] 9^a^ série ou mais [RP = 1,8] Sexo do bebê feminino [RP = 1,3]	Área (urbana/rural); fornecimento de água; renda *per capita*; nº de moradores; informação pré-natal sobre AM; nº de consultas de pré-natal; tipo de parto; peso ao nascer; trabalho materno; ser atendido por ESF
Carvalhaes et al^7^ (2007)	Botucatu, SP, 2004	380 bebês < 4 m/AMAMUNIC	Regressão logística hierarquizada	Moderado	38,0% (< 4 m)	Interrupção do AME	Dificuldade em iniciar o aleitamento materno [OR = 1,57] Uso de chupeta [OR = 2,63]	Escolaridade materna; paridade; trabalho materno; licença maternidade; financiamento do hospital; tipo de parto; peso ao nascer
Leone et al^21^ (2012)	São Paulo, SP, 2008	724 bebês < 6 m/AMAMUNIC	Regressão logística	Moderado	39,6% (< 6 m)	Interrupção do AME	Trabalho materno [OR = 2,11] Idade da criança em dias [OR = 1,01] Uso de chupeta [OR = 3,02]	Escolaridade materna; tipo de parto; nascimento em HAC; peso ao nascer; sexo do bebê; AM na 1^a^ hora de vida
Martins et al^23^ (2011)	Feira de Santana, BA, 2004 a 2005	1.309 bebês < 1 m/visita domiciliar	Regressão logística	Moderado	59,3% (ao 1º m)	Interrupção do AME	Escolaridade materna: ≤ ensino médio [OR = 1,35] Primíparidade [OR = 1,41] Renda < 1 salário mínimo [OR = 1,27] Falta de orientação em AM no hospital [OR = 1,53] Sentir-se cansada [OR = 1,18]	Idade materna; tipo de parto
Nascimento et al^28^ (2010)	Joinville, SC, 2005	1.470 bebês < 6 m/AMAMUNIC	Regressão Poisson	Moderado	71,2% (1º mês) 61,0% (2º mês) 46,3% (3º mês) 44,2% (4º mês) 31,3% (5º mês) 13,3% (6º mês)	Interrupção do AME	Escolaridade materna < 12 anos [RP = 1,59] Idade da criança ≥ 90 dias [RP = 1,53] Uso de chupeta [RP = 1,69]	Idade materna; paridade; nascimento em HAC; tipo de parto; sexo do bebê; peso ao nascer; financiamento da atenção primária; profissional que assistiu a criança; trabalho materno
Neves et al^29^ (2014)	252 municípios das regiões N e NE, 2010	9.060 bebês < 6 m/Chamada Neonatal	Regressão Poisson	Moderado	39,9% (< 6 m)	AME	Local da residência: capital [RP = 1,17] Idade materna (< 20 anos): 20-34 anos [RP = 1,14] ≥ 35 anos [RP = 1,28] Idade da criança (0 mês): 1 mês [RP = 0,77] 2 meses [RP = 0,68] 3 meses [RP = 0,53] 4 meses [RP = 0,37] 5 meses [RP = 0,16] AM na 1ª hora de vida [RP = 1,16]	Escolaridade materna; cor da pele; realização de pré-natal; financiamento do pré-natal; orientação pré-natal sobre AM; tipo de parto; financiamento da maternidade; alojamento conjunto; sexo do bebê; peso ao nascer; visita recente de agente de saúde
Parizoto et al^30^ (2009)	Bauru, SP, 2006	509 bebês < 6 m/AMAMUNIC	Regressão logística	Moderado	24,2% (< 6 m) 3,9% (aos 6 m)	Interrupção do AME	Uso de chupeta [OR = 2,03]	Idade materna; escolaridade materna paridade; tipo de parto; financiamento da maternidade; peso ao nascer; trabalho materno
Pereira et al^31^ (2010)	Rio de Janeiro, RJ, 2007	1.029 bebês < 6 m/clientela de 27 unidades básicas	Regressão de Poisson hierarquizada	Moderado	58,1% (< 6 m)	AME	Cor da pele branca [RP = 1,20] Morar com companheiro [RP = 1,72] Amamentação pregressa [RP = 1,27] Informação pré-natal sobre AM [RP = 1,27] AME na alta hospitalar [RP = 2,01] Orientação sobre AM em grupo [RP = 1,14] Orientação sobre como colocar o bebê para mamar [RP = 1,20] Idade do bebê em meses [RP = 0,83]	Idade materna; paridade; nº de bens na residência; nº de consultas de pré-natal; nascimento em HAC (ou em processo de acreditação); tipo de parto; peso ao nascer; sexo do bebê; trabalho materno; tipo da unidade básica de saúde; orientações sobre livre demanda, ordenha, período de ame e não uso de mamadeira; satisfação materna com o apoio recebido na unidade básica para amamentar;
Queluz et al^34^ (2012)	Serrana, SP, 2009	275 bebês < 6 m/AMAMUNIC	Regressão logística	Moderado	29,8% (< 6 m)	Interrupção de AME	Trabalho materno fora de casa sem licença maternidade [OR = 3,08] não trabalha fora [OR = 2,26]	Idade materna; paridade; escolaridade materna; nascimento em HAC; tipo de parto; peso ao nascer; uso de chupeta; financiamento da puericultura
Rito et al^38^ (2013)	Rio de Janeiro, RJ, 2007-2008	4.092 bebês < 6 m/clientela de 56 unidades básicas	Regressão de Poisson	Moderado	47,6% (< 6 m)	AME	Desempenho na Iniciativa Unidade Básica Amiga da Amamentação: superior [RP = 1,34] intermediário [RP = 1,17] Unidade básica de saúde [RP = 1,10] Não trabalho materno [RP = 1,75] Idade da criança em dias [RP = 0,99]	Ter recebido orientação/ajuda do hospital para amamentar
Vannuchi et al^44^ (2005)	Londrina, PR, 2002	988 bebês < 6 m/AMAMUNIC	Regressão logística	Moderado	21,0% (< 6 m)	Interrupção do AME	Primiparidade [OR = 1,63] Uso de chupeta [OR = 2,23] Acompanhamento ambulatorial em rede pública [OR = 2,08]	Peso ao nascer; idade do bebê em dias
Venâncio et al^46^ (2002)	84 municípios do Estado de São Paulo, 1998	11.481 bebês < 6 m/AMAMUNIC	Regressão logística	Moderado	De 0% (em 10 cidades) a 54,0% (em 1 cidade)	Interrupção do AME	Idade materna adolescente [OR = 1,20] Escolaridade materna (≥ 13 anos) 9-12 anos [OR = 1,54] 5-8 anos [OR = 1,94] Até 4 anos [OR = 2,28] Primiparidade [OR = 1,27] Nascimento em HAC [OR = 1,49] Município com HAC [OR = 2,28]	Trabalho materno
Venâncio et al^47^ (2006)	111 municípios do Estado de São Paulo, 1999	34.345 bebês < 6 m/AMAMUNIC	Regressão logística multinível	Moderado	13,9% (< 6 m); 4,0% (aos 6 m)	AME	Escolaridade materna (< primário) primário completo [OR = 1,15] secundário incompleto [OR = 1,18] secundário completo [OR = 1,58] superior ≥ incompleto [OR = 1,91] Idade materna (11 a 17 anos): 18-19 anos [OR = 1,17] 20-24 anos [OR = 1,43] 25-29 anos [OR = 1,52] 30-34 anos [OR = 1,52] ≥ 35 anos [OR = 1,22] Multiparidade [OR = 1,41] Peso ao nascer (< 1.500 g): 2.000-2.499 g [OR = 1,29] 2.500-2.999 g [OR = 1,52] ≥ 3.000 g [OR = 1,73] Sexo da criança feminino [OR = 1,12] Financiamento privado da puericultura [OR = 1,10] Municípios com 4 a 5 ações pró-AM [OR = 1,53]	Município de residência; tamanho da população; índice de desenvolvimento humano (SES/SP); tipo de parto; nascimento em HAC; trabalho materno
Audi et al^2^ (2003)	Itapira, SP, 1999	346 bebês < 6 m/AMAMUNIC	Regressão logística	Fraco	64,8% (ao 1º mês) 9,6% (entre o 4^o^ e 6^o^ mês)	Interrupção do AME	Tipo de parto (cesariano) [OR = 1,78] Uso de chupeta (sim) [OR = 4,41]	Local de moradia; idade materna; escolaridade materna; paridade; financiamento do hospital; peso ao nascer; trabalho materno; licença maternidade; financiamento da puericultura
Damião^10^ (2008)	Rio de Janeiro, RJ, 1998 e 2000	2.459 bebês < 4 m/AMAMUNIC	Regressão logística	Fraco	22,7% (< 4 m)	AME	Idade materna em anos [OR = 1,02] Escolaridade materna 3^o^ grau completo [OR = 1,93] Trabalho materno [OR = 0,59] Idade da criança em dias [OR = 0,99]	Paridade; nascimento em HAC; peso ao nascer
Fernandes et al^15^ (2012)	Rio de Janeiro, RJ, 2005-2008	592 bebês < 1 m/clientela de 4 unidades básicas	Regressão logística	Fraco	Cerca de 75,0% (< 1 m)	Interrupção do AME	Obesidade pré-gestacional [OR = 2,14] Sobrepeso com excessivo ganho de peso gestacional (GPG) [OR = 2,29] Obesidade com insuficiente GPG [OR = 3,10] Obesidade com excessivo GPG [OR = 3,33] GPG excessivo [OR = 1,50]	Educação materna; fumo na gestação; nº consultas pré-natais; tipo de parto; peso ao nascer; apoio social; rede social
França et al^16^ (2007)	Cuiabá, MS, 2004	275 bebês < 6 m/AMAMUNIC	Regressão logística	Fraco	34,5% (< 6 m)	Interrupção do AME	Idade materna (≥ 35 anos): < 20 anos [OR = 3,54] 20 a 34 anos [OR = 3,13] Escolaridade materna 1° e 2° graus [OR = 2,31] Primiparidade [OR = 2,20] Uso de chupeta [OR = 3,26]	Nascer em hospital com Banco de Leite; tipo de parto; trabalho materno; financiamento da atenção primária
Gusmão et al^18^ (2013)	Porto Alegre, RS, 2009	341 bebês < 6 m filhos de mães adolescentes/Inquérito domiciliar	Regressão Poisson hierarquizada	Fraco	37,8% (< 6 m)	AME	Escolaridade materna: ensino médio [RP = 1,53] Multiparidade [RP = 1,57] Idade da criança em meses [RP = 0,76]	Idade materna; Cor da pele; Escolaridade da avó materna; Frequenta escola; Situação conjugal; Possui renda; Classe social; Financiamento do pré-natal; Nº de consultas de pré-natal; Nascimento em HAC; Tipo de parto; Desejo pela gravidez; Atitude do parceiro; Reação da família; Indicadores emocionais; Expectativa relativa ao futuro; Autovalorização; Sofrimento psíquico; Peso ao nascer; Apgar 5º minuto; Sexo do bebê; Cuidador do bebê; Grau de dificuldade para cuidar; Percepção da saúde do bebê
Vieira et al^51^ (2010)	Feira de Santana, BA, 2004 a 2005	1.309 bebês com um mês de vida/entrevistas	Regressão logística	Fraco	59,3% (ao final do 1º mês)	Interrupção do AME	Experiência prévia com amamentação ausente [OR = 1,24] Horários pré-estabelecidos para amamentar [OR = 1,42] Uso de chupeta [OR = 1,53] Presença de fissura mamilar [OR = 1,25]	Peso ao nascer; AM na primeira hora de vida

AME: Aleitamento Materno Exclusivo; ESF: Estratégia de Saúde da Família; AM: aleitamento materno; HAC: Hospital Amigo da Criança

**Tabela 2:** Estudos de coorte incluídos sobre fatores associados ao aleitamento materno exclusivo.

Autor e ano de publicação	Local e ano de realização	Amostra (n) e fonte de dados	Análise estatística	Escore de Qualidade	Prevalência AME (faixa etária)	Desfecho	Fatores associados ao AME	Fatores avaliados sem significância estatística
Chaves et al^9^ (2007)	Hospital Municipal de Itaúna, MG, 2003	238 bebês acompanhados até 6 m	Regressão de Cox	Forte	62,6% (1 mês); 19,5% (4 meses); 5,3% (6 meses)	Interrupção do AME	Intenção de amamentar (> 24 meses): < 12 meses [RR = 1,67] 12 a 23 meses [RR = 1,74] Peso ao nascer > 2.500 g [RR = 1,92] Uso de chupeta [RR = 1,49]	Saneamento básico; cor da pele; escolaridade materna; estado civil; paridade; renda; informação sobre a técnica de AM; uso de álcool ou tabaco; plano de saúde; nº consultas de pré-natal; idade gestacional; sexo do bebê; trabalho materno; licença maternidade; apoio familiar; tempo até a primeira mamada; intercorrências após o parto
Demétrio et al^11^ (2012)	Laje e Mutuípe, BA, 2005 a 2008	531 bebês acompanhados até 6 m /AMACOMP	Regressão de Cox	Forte	74,7 dias (mediana)	Interrupção do AME	Residência urbana [HR = 1,61] Não realização de pré-natal [HR = 2,73]	Condição de moradia; idade materna; cor da pele; escolaridade materna; estatura materna; estado nutricional antropométrico; tipo de parto; peso ao nascer; idade gestacional; sexo do bebê; trabalho materno
Mascarenhas et al^24^ (2006)	Pelotas, RS, 2002 a 2003	940 bebês acompanhados até 3 m	Regressão logística hierarquizada	Forte	39,0% (aos 3 meses)	Interrupção do AME antes dos 3 m	Renda familiar (> 6 Salários Mínimos - SM): 1,1 a 3 SM [OR = 1,60] Escolaridade paterna (≥ 9 anos): 0 a 4 anos [OR = 1,61] Trabalho materno [OR = 1,76] Uso de chupeta [OR = 4,25]	Cor da pele; idade materna; escolaridade materna; paridade; nº consultas de pré-natal; tabagismo na gravidez; peso ao nascer; idade gestacional; sexo do bebê
Santo et al^40^ (2007)	Hospital das Clínicas (Porto Alegre, RS), 2003	220 bebês com até 6 m (com peso ao nascer ≥ 2.500 g)	Regressão de Cox	Forte	54,0% (no 1º mês); 6,6% (aos 6 m)	Interrupção do AME	Idade materna adolescente [HR = 1,48] < 6 consultas pré-natal (< 6 consultas) [HR = 1,60] Uso de chupeta [HR = 1,53] Nº avaliações negativas de pega do peito na maternidade [HR = 1,29]	Cor da pele; escolaridade materna; paridade; situação conjugal; duração do AM do filho anterior; viver com a avó da criança; informações pré-natais sobre AM; participar de grupo de gestantes no pré-natal; lesão mamilar no pós-parto; nº de avaliações negativas de posição ao peito na maternidade
Silva et al^43^ (2008)	Pelotas, RS, 2002 a 2003	951 bebês acompanhados até um mês de idade.	Regressão logística hierarquizada	Forte	60,0% (ao 1º mês de vida)	Interrupção do AME no 1º mês de vida	Escolaridade paterna (≥ 9anos) 5 a 8 anos [OR = 1,31] 0 a 4 anos [OR = 1,63] Idade paterna (maior que 35 anos) 20 a 34 anos [OR = 1,45] menos de 20 anos [OR = 1,43] Uso de chupeta [OR = 2,45]	Cor da pele; nº de consultas no pré-natal; tabagismo na gravidez; nascer em Hospital Amigo da Criança; peso ao nascer; sexo do bebê; trabalho materno
Vieira et al^52^ (2014)	Feira de Santana, BA, 2004 a 2005	1.344 crianças acompanhadas até 6 m de idade	Regressão de Cox hierarquizada	Forte	89 dias (mediana)	Interrupção do AME	Escolaridade materna ≤ 8anos [HR = 1,34] < 6 consultas pré-natal [HR = 1,48] Financiamento público do pré-natal [HR = 1,34] Nascer em Hospital Amigo da Criança [HR = 0,85] Orientação sobre AM no hospital [HR = 0,80] Companheiro favorável à amamentação [HR = 0,62] Trabalho materno [HR = 1,73] Mãe limitar as mamadas noturnas [HR = 1,58] Fissura mamilar [HR = 2,54] Uso de chupeta [HR = 1,40]	Cor da pele; idade materna; paridade; experiência prévia com AM; mãe morar com pai da criança; mãe ter participado de grupo de pré-natal sobre AM; tipo de parto; AM na primeira hora de vida; sexo do bebê
Bueno et al^5^ (2003)	Hospital Universitário em São Paulo, SP, 1998 a 1999	383 bebês acompanhados até 6 m	Regressão de Cox	Fraco	23 dias (mediana); 38,5% (1 m); 13,8% (3 m); 1,6% (6 m).	Interrupção do AME	Idade materna ≤ 25 anos [HR = 1,22] Escolaridade materna (superior): ensino fundamental [HR = 2,13] ensino médio [HR = 1,78] Sexo feminino do bebê [RR = 1,22]	Morar na favela; cor da pele; paridade; situação conjugal; número de bens de consumo; número de consultas de pré-natal; hábito de fumar; tipo de parto; peso ao nascer

AM: aleitamento materno; AME: aleitamento materno exclusivo

A maior parte dos estudos foram conduzidos em cidades e abrangeram 77.866 crianças. Quanto às regiões brasileiras, 14 estudos foram realizados no Sudeste do Brasil, seis no Sul, cinco no Nordeste, um no Centro-Oeste e um na região Norte. A revisão sistemática abrangeu estudos conduzidos entre 1998 e 2010 (Tabelas 1 e 2).

A prevalência pontual do aleitamento materno exclusivo aos seis meses variou de 3,9% em Bauru^30^ a 8,5% em Pernambuco, ambas em 2006.^6^ Quanto ao indicador da OMS, a prevalência de aleitamento materno exclusivo em crianças menores de seis meses de vida, resultante de inquérito dessa população, variou de 0% em 10 cidades do estado de São Paulo em 1998^46^a 58,1% na cidade do Rio de Janeiro em 2007^31^ (Tabelas 1 e 2).

Os fatores associados ao aleitamento materno exclusivo foram organizados em níveis hierarquizados (Tabela 3), sendo os mais frequentemente explorados (mais de um quinto dos 27 estudos): local de residência, cor da pele, idade e escolaridade maternas, paridade, situação conjugal, número de consultas pré-natais, nascimento em Hospital Amigo da Criança, tipo de parto, peso ao nascer, sexo do recém-nascido, trabalho materno, idade da criança, financiamento da unidade de atenção primária à saúde da criança e uso de chupeta.

**Tabela 3:** Fatores investigados quanto à associação com o aleitamento materno exclusivo, organizados por nível hierarquizado, frequência de utilização e número de vezes em que se associaram de forma estatisticamente significativa ao aleitamento materno exclusivo.

Fator e nível	Estudos	Associação	Fator e nível	Estudos	Associação	Fator e nível	Estudos	Associação	Fator e nível	Estudos	Associação

Contextual	n	n	Intermediário distal	N	n	Intermediário proximal	n	n	Proximal	n	n
Total de ações em aleitamento materno	1	1	Estado nutricional antropométrico	2	1	Nascer em Hospital Amigo da Criança	12	1	Trabalho materno	20	6
Tamanho da população	1	0	Desejo pela gravidez	1	0	Nascer em hospital com Banco de Leite Humano	1	0	Licença maternidade	4	1
Índices compostos de população	2	0	Tabagismo	4	0	Tipo de financiamento da maternidade ou hospital	4	0	Cansaço ou indicadores emocionais	2	1
Local de residência	7	3	Alcoolismo	1	0	Orientação sobre aleitamento materno na maternidade ou atendimento profissional	4	2	Conhecimentos sobre a técnica de aleitamento	1	0
Saneamento básico	3	0	Consultas pré-natais	12	3	N^o^ avaliações negativas de pega do peito	1	1	Dificuldades em amamentar	3	2
Distal			Informações sobre aleitamento materno no pré-natal	4	1	N^o^ avaliações negativas de posição ao peito	1	0	Apoio familiar	3	1
									Cuidador do bebê	1	0
Cor da pele ou raça	10	1	Participação de grupo de pré-natal	2	0	Alojamento conjunto	1	0	Viver com a avó da criança	1	0
Idade materna	20	8	Financiamento do pré-natal	4	1	Intenção de amamentar	1	1	Idade da criança	8	8
Escolaridade materna	23	11				Tipo de parto	19	2	Saúde da criança	2	0
Paridade	19	6				Tempo até a primeira mamada	5	2	Uso de chupeta	16	15
Experiência prévia com amamentação	4	2				Aleitamento materno exclusivo na alta hospitalar	1	1	Financiamento da atenção primária em saúde	6	2
Situação conjugal	6	1				Peso ao nascer	21	3	Tipo Unidade Básica	4	1
Idade paterna	1	1				Idade gestacional	4	0	Orientação sobre aleitamento em grupo	1	1
Escolaridade paterna ou da avó	3	2				Intercorrências imediatas após o parto e Apgar	2	0	Orientação sobre pega ou posição	1	1
Renda familiar	5	2				Sexo do recém-nascido	14	3	Outras orientações sobre aleitamento	1	0
Número de pessoas na casa	1	0							Satisfação das mães com o apoio recebido	1	0
Número de bens na residência	2	0							Acompanhamento em Unidade Básica Amiga da Amamentação	2	2

Os fatores mais frequentemente associados ao aleitamento materno exclusivo (fatores investigados em pelo menos seis estudos e que apresentaram associação em pelo menos um terço dos estudos onde foram investigados) foram (segundo a categoria associada positivamente ao desfecho): local de residência (residência na capital, na região metropolitana ou no meio rural), idade materna intermediária, escolaridade materna crescente, ausência de trabalho materno, idade da criança (decrescente), não uso de chupeta e financiamento da atenção primária à saúde (privado) (Tabelas 1, 2 e 3).

Os estudos elencaram, ao total, 36 fatores que estiveram associados ao aleitamento materno exclusivo*,* 11 classificados como distais, quatro como intermediários distais*,* nove como intermediários proximais e 12 como proximais (Tabela 3).

Dos estudos selecionados, oito utilizaram modelo teórico hierarquizado para identificar os fatores associados ao aleitamento materno exclusivo antes de iniciar a modelagem estatística, sendo que somente um levou em conta variáveis contextuais^47^ (Tabelas 1 e 2).

Baseado nos fatores elencados nas análises dos 27 estudos selecionados, foi construído modelo teórico hierarquizado dos fatores associados ao aleitamento materno exclusivo. Alguns foram constituídos do agrupamento de fatores semelhantes, como em “dificuldades em amamentar”, que agrupou as variáveis: fissura mamilar, horários pré-estabelecidos para amamentar e dificuldades de pega ou posição. Da mesma forma, foram agrupados como “indicadores emocionais” as variáveis: autovalorização e sofrimento psíquico maternos (Figura 2).

**Figura 2:** Modelo teórico hierarquizado dos fatores associados ao aleitamento materno exclusivo.

## DISCUSSÃO

A revisão sistemática de estudos epidemiológicos brasileiros mostrou produção relevante de estudos a partir do final da década de 1990 tendo o aleitamento materno exclusivo como desfecho, os quais foram conduzidos principalmente na região Sudeste do Brasil. A maior parte dos estudos selecionados para esta revisão apresentou qualidade moderada, e apenas um quarto dos artigos teve desenho longitudinal. Portanto, a evidência dos fatores associados ao aleitamento materno exclusivo em menores de seis meses no Brasil encontrada na presente revisão pode ser considerada como moderada.

Foi elevado o número de variáveis elencadas nos estudos epidemiológicos utilizadas para explicar a duração do aleitamento materno exclusivo, sendo que a discussão dos achados desta revisão sistemática foi pautada pela organização das variáveis em níveis hierarquizados. Devido à diversidade de cenários e fatores investigados, o uso de medidas sumárias de associação derivadas de técnicas de meta-análise não foi considerado procedente.

Entre os fatores distais, o local de residência foi a variável contextual mais investigada, e os resultados foram discordantes, ora o meio urbano,^6,29^ ora o meio rural^11^ tendo se associado ao aleitamento materno exclusivo. A maioria dos fatores distais parece representar fatores socioeconômicos maternos. A escolaridade materna foi o fator mais amplamente investigado, quase a metade dos estudos tendo observado associação entre escolaridade materna e aleitamento materno exclusivo, e os achados foram unânimes: a baixa escolaridade associou-se à interrupção do aleitamento materno exclusivo. Nos estudos epidemiológicos, o gradiente socioeconômico em geral se reproduz em um gradiente de saúde.^22^ As pesquisas nacionais sobre aleitamento materno também reproduzem essas diferenças, em que mães com maior escolaridade amamentam exclusivamente por mais tempo.^d^

A variável “cor da pele ou raça”, por sua vez, pode representar costumes, normas e tradições sociais,^27^ além de renda^8^ e relações sociais.^e^ Considerando as pesquisas nacionais, mães brancas amamentaram exclusivamente por mais tempo,^b^ porém apenas um estudo encontrou associação entre a cor da pele branca e maiores prevalências de aleitamento materno exclusivo.^31^

A idade materna e a paridade podem representar a experiência com o aleitamento materno.^31,51^ Todos os estudos que as investigaram, observaram associação entre maior paridade e aleitamento materno exclusivo.^16,18,23,44,46,47^ Quanto à idade materna, as idades intermediárias parecem ser protetoras para o aleitamento materno exclusivo, pois tanto mães adolescentes o interrompem mais precocemente^5,29,40,46,47^ quanto aquelas com 35 anos ou mais.^6,10,16^

Considerando os fatores intermediários distais, relativos à gestação, o número de consultas pré-natais foi a variável mais frequentemente investigada. Os três estudos que encontraram associação entre esta variável e o desfecho indicaram o baixo número de consultas pré-natais como fator de risco para o aleitamento materno exclusivo. Santo et al^40^ e Vieira et al^52^ consideram que a baixa adesão ao pré-natal pode representar mulheres que tenham menos cuidado com sua saúde; já Demétrio et al^11^ consideram que essa baixa adesão pode refletir baixo acesso a fontes de informação sobre aleitamento materno.

O atendimento público ou privado perpassa todos os níveis hierarquizados avaliados: pré-natal (nível intermediário distal), parto (intermediário proximal) e puericultura (proximal). A assistência primária privada se associou ao desfecho em três de 10 estudos.^44,47,52^ Essa variável pode representar tanto o acesso aos serviços de saúde, quanto a situação socioeconômica materna, pois o acesso aos serviços de saúde pode ser determinado por variáveis mais distais, como cor da pele, gênero, escolaridade e renda.^36^

Entre os fatores intermediários proximais, o peso ao nascer foi o fator mais amplamente utilizado, sendo encontrada associação positiva entre crianças com peso ao nascer adequado e aleitamento materno exclusivo em três dos 21 estudos que o investigaram.^9,44,47^ Isso pode ser explicado pelo fato de crianças com baixo peso ao nascer estarem mais propensas a ficar mais tempo internadas em unidade neonatal, passando, assim, mais tempo separadas de suas mães.^41^ Além disso, essas crianças podem ter mais dificuldades em iniciar ou manter o aleitamento materno, pois tanto a frequência, quanto a pressão da sucção aumentam conforme aumenta a idade gestacional e peso do recém-nascido.^25^

A idade gestacional, por sua vez, foi um indicador pouco utilizado nos estudos, pois podem ocorrer diferenças ou vieses nas classificações dessa variável.^42^ Apesar de nenhum estudo ter encontrado associação entre essa variável e o desfecho, sugere-se que esta seja mantida nos estudos.

Outro fator amplamente utilizado nos estudos foi o tipo de parto, porém, apenas dois estudos encontraram associação entre o parto normal e maior prevalência de aleitamento materno exclusivo.^1,2^ O parto normal contribui para o início oportuno do aleitamento materno,^3^ sendo possível supor que também possa propiciar a sua manutenção na modalidade exclusiva. Outra hipótese seria a possível relação entre características socioeconômicas e acesso aos serviços públicos de saúde,^17^ já que tanto o parto normal,^13^ quanto o início precoce da amamentação são mais praticados nesses serviços.^3^

Entre os fatores intermediários proximais estudados, os que aferem as orientações recebidas no hospital^23,52^ (associação positiva com o desfecho), as dificuldades para amamentar durante a internação hospitalar^7^ (associação negativa) e o aleitamento materno exclusivo na alta hospitalar^31^ (associação positiva) podem ser os mais adequados para avaliar aspectos relacionados ao peri-parto que venham a determinar a duração do aleitamento materno exclusivo.

A variável sexo do bebê foi utilizada em 14 estudos, sendo que dois^6,47^ encontraram associação positiva entre sexo feminino, e um entre sexo masculino^5^ e aleitamento materno exclusivo. Maior prevalência do aleitamento materno entre as meninas foi observada nas capitais de todo o Brasil;^b^ contudo, não está claro se essa maior prevalência deveu-se a algum aspecto cultural, como a crença de que meninos precisam de maior aporte nutricional por meio de outros alimentos além do leite materno.^32,47^

Em relação aos fatores proximais considerados, o uso de chupeta foi o fator mais fortemente associado à interrupção do aleitamento materno exclusivo.^2,7,9,16,21,24,28,30,43,44,51,52^ O uso de chupeta pode levar à redução da frequência de amamentação, interferindo na demanda ao seio, e, possivelmente, alterando a dinâmica oral do bebê.^50^ Um estudo brasileiro concluiu que, além de a relação causal entre uso de chupeta e interrupção do aleitamento não estar clara (não se sabe se o uso de chupeta é um marcador da interrupção do aleitamento materno, ou se é uma causa do mesmo), o processo do uso de chupetas é dinâmico, com crianças iniciando ou interrompendo o uso de chupeta ao longo do período.^50^ Em estudo randomizado no Canadá,^19^ os autores observaram que o uso de chupeta pode ser marcador da interrupção do aleitamento materno ou de baixa motivação para amamentar, ao invés de ser causa da interrupção do aleitamento materno.

O trabalho materno foi uma variável amplamente utilizada nos estudos,^1,2,6,7,9-11,16,21,24,28,30,31,34,38,43-47,52^ e nos seis estudos que encontraram associação estatisticamente significativa, a mesma se mostrou negativamente associada ao desfecho. Porém, esta variável deve ser investigada levando em conta se a mãe está ou não em licença maternidade.^51^ Mães que trabalham fora com licença maternidade teriam melhores condições para manter o aleitamento materno exclusivo durante o período da licença.

A maior parte dos estudos baseados em inquéritos não levaram em consideração a idade da criança, mas a probabilidade de ser amamentada exclusivamente diminui à medida que aumenta a idade da criança. Todos os estudos que utilizaram essa variável encontraram associação entre idade decrescente (ou menor idade) da criança e aleitamento materno exclusivo.^1,10,18,21,28,29,31,44^

Entre todas as variáveis consideradas proximais, as que avaliam o acesso à informação ou orientação sobre aleitamento materno que as mulheres recebem nos serviços de atenção primária em saúde, poderiam ser aquelas mais diretamente associadas ao aleitamento materno exclusivo. Contudo, apenas Pereira et al^31^ utilizaram essa variável, observando que orientações em grupo e sobre posição e pega do bebê no peito se associaram a maior prevalência do aleitamento materno exclusivo.

Avaliando as estratégias de modelagem estatística, pouco menos de um quarto dos estudos compreendidos nesta revisão adotaram modelo teórico prévio às análises, organizando as variáveis em níveis hierarquizados.^1,6,7,18,24,31,43,52^ Apesar de dispensável, construir esse modelo conceitual é importante, pois requer conhecimento prévio sobre os fatores sociais e biológicos associados ao desfecho, auxiliando a estabelecer uma ordem de entrada lógica das variáveis no modelo baseado na hierarquia de fatores e não considerando apenas critérios puramente estatísticos.^49^ Nesse intuito, foi proposto modelo teórico hierarquizado, compreendendo os fatores identificados nos estudos desta revisão sistemática, que pode auxiliar no planejamento da coleta de dados e na estratégia de modelagem estatística dos estudos epidemiológicos relacionados ao aleitamento materno exclusivo.

As políticas públicas de promoção, proteção e apoio ao aleitamento materno adotadas no Brasil desde a década de 1980 têm contribuído para o aumento da duração mediana do aleitamento materno e de sua modalidade exclusiva em todo o País.^35^ Essas políticas, contudo, não podem ser consideradas como atributo do indivíduo: ter um parto em Hospital Amigo da Criança ou em maternidade com Banco de Leite Humano pode depender do contexto em que a mulher vive, bem como de seu acesso a esses serviços.

Além disso, os contextos locais dentro de cada cidade (distritos, bairros, vizinhanças) podem variar: na cidade do Rio de Janeiro, e.g., há grande variação na adoção dos 10 passos para o sucesso da amamentação (preconizadas pela Iniciativa Unidade Básica Amiga da Amamentação) entre as unidades da rede primária de saúde.^37^ Devem ser levadas em conta, também, as disparidades entre as unidades de saúde públicas e privadas, como as observadas na adoção da amamentação na primeira hora de vida nos hospitais (preconizada pela Iniciativa Hospital Amigo da Criança).^3^

Tendo em vista esse possível efeito de contexto, é plausível que nutrizes que residam nas mesmas regiões ou municípios (incluindo distritos, bairros ou unidades censitárias) compartilhem fatores sociais e econômicos (fatores contextuais) que influenciem na duração do aleitamento materno exclusivo, e.g., normas e atitudes em relação ao aleitamento materno; a organização e acesso aos serviços de atenção primária de saúde de seu bairro; e o nível de adoção das ações e políticas de promoção, proteção e apoio ao aleitamento materno. De fato, variáveis como os índices socioeconômicos compostos e o número de ações pró-aleitamento materno existentes em determinada região já foram utilizadas para a avaliação dos fatores associados ao aleitamento materno exclusivo.^47^

Foram identificados e descritos grande número de fatores utilizados em estudos epidemiológicos analíticos e sua direcionalidade na associação com o aleitamento materno exclusivo, observando-se a frequência com que são utilizados e a heterogeneidade de categorias e pontos de corte. Ao invés de definir o efeito de cada um dos fatores identificados na revisão sistemática por meio de meta-análise, optou-se por discutir os mesmos segundo um modelo hierarquizado teórico.

Algumas recomendações referentes aos achados deste estudo incluem a realização de mais estudos nas regiões Norte e Centro-Oeste do País, bem como o incentivo à produção acadêmica sobre fatores pouco explorados na associação com o aleitamento materno exclusivo.

O uso de modelo teórico conceitual prévio às análises estatísticas, dando preferência à organização das variáveis de forma hierarquizada em relação à proximidade com o desfecho, pode auxiliar a escolha das variáveis que serão incluídas nos estudos e avaliar a intermediação dos blocos de variáveis mais proximais em relação às mais distais.

Sugere-se que futuros estudos considerem variáveis de contexto para investigar a associação com o aleitamento materno exclusivo, uma vez que a inclusão de variáveis contextuais concomitante aos modelos multinível é uma estratégia útil para a adequação desses modelos.^12^ A realização de estudos que contemplem a triangulação de métodos qualitativos e quantitativos^26^ para a compreensão da relação de alguns fatores com o aleitamento materno exclusivo poderia contribuir também para uma melhor compreensão da temática.

A principal limitação dessa revisão sistemática foi o viés de seleção, pois não foram incluídos resumos publicados em anais de congressos, a chamada “literatura cinzenta”.^33^ Outra limitação é a possibilidade de estudos relevantes não terem sido encontrados pela estratégia de busca utilizada. A possível subjetividade dos autores na avaliação e seleção dos artigos foi minimizada pela busca independente da literatura, pelo preenchimento de formulário padronizado e pela avaliação da qualidade dos artigos selecionados para a revisão.

Concluindo, o estudo dos determinantes do aleitamento materno exclusivo é de vital importância para a saúde pública, e os estudos epidemiológicos vêm cumprindo papel importante para a compreensão desse tema no Brasil. No entanto, o surgimento de novas e mais sofisticadas ferramentas estatísticas, bem como a crescente complexidade dos modelos explicativos e os efeitos de contexto dos fatores associados ao aleitamento materno exclusivo, trazem um novo desafio aos estudiosos do tema: o uso criterioso desses recursos e a divulgação dos resultados de forma clara e propositiva, direcionada à elaboração e aperfeiçoamento de políticas públicas de promoção, proteção e apoio ao aleitamento materno que repercutam na saúde e bem-estar da população.

## Footnotes

a Ministério da Saúde, Secretaria de Atenção à Saúde, Departamento de Ações Programáticas e Estratégicas. II Pesquisa de prevalência de aleitamento materno nas capitais brasileiras e Distrito Federal. Brasília (DF): Ministério da Saúde; 2009. (Série C. Projetos, programas e relatórios).

b Ministério da Saúde. PNDS 2006: pesquisa nacional de demografia e saúde da criança e da mulher. Brasília (DF): Ministério da Saúde; 2008.

c World Health Organization. Indicators for assessing infant and young child feeding practices. Part 1: definitions. Conclusions of a consensus meeting held 6-8 November 2007 in Washington, DC, USA. Geneva: World Health Organization; 2007.

d Ministério da Saúde. PNDS 2006: Pesquisa Nacional de Demografia e Saúde da Criança e da Mulher. Brasília (DF). Secretaria de Ciência, Tecnologia e Insumos Estratégicos; 2008. 326p.

e Instituto Brasileiro de Geografia e Estatística. Características étnico-raciais da população: um estudo das categorias de classificação de cor ou raça: 2008. Rio de Janeiro (RJ): Instituto Brasileiro de Geografia e Estatística; 2011.
